# Visually Inexperienced Chicks Exhibit Spontaneous Preference for Biological Motion Patterns

**DOI:** 10.1371/journal.pbio.0030208

**Published:** 2005-06-07

**Authors:** Giorgio Vallortigara, Lucia Regolin, Fabio Marconato

**Affiliations:** **1**Department of Psychology and B.R.A.I.N. Centre for Neuroscience, University of TriesteTriesteItaly; **2**Department of General Psychology, University of PaduaPadovaItaly; Istituto di NeurofisiologiaItaly

## Abstract

When only a small number of points of light attached to the torso and limbs of a moving organism are visible, the animation correctly conveys the animal's activity. Here we report that newly hatched chicks, reared and hatched in darkness, at their first exposure to point-light animation sequences, exhibit a spontaneous preference to approach biological motion patterns. Intriguingly, this predisposition is not specific for the motion of a hen, but extends to the pattern of motion of other vertebrates, even to that of a potential predator such as a cat. The predisposition seems to reflect the existence of a mechanism in the brain aimed at orienting the young animal towards objects that move semi-rigidly (as vertebrate animals do), thus facilitating learning, i.e., through imprinting, about their more specific features of motion.

## Abstract 2

Taking advantage of the spontaneous imprinting behaviour of newly hatched chicks, Giorgio Vallortigara and colleagues study their innate ability to distinguish biological motion.

## Introduction

It has long been known in the literature on imprinting [1,2], and indeed in studies of mammals, including human infants [3], that moving objects are more likely to evoke a response than are stationary objects. What is unknown, however, is whether learning plays a part in the formation of this preference. Consider the case of filial imprinting. By looking at the ethological literature (review in [4]), one finds the general assertion that object motion facilitates imprinting. However, no one has checked whether all types of motion are identically effective or if animals are especially sensitive to particular types of motion. The problem, of course, is that it is difficult to disentangle the stationary visual characteristics of an object (its shape, texture, colour, and brightness) from the dynamic aspects (its motion). We used point-light displays to solve the problem.

When a biological creature, such as a hen, travels about its environment, its limbs and torso move in characteristic synchrony. Johansson [5] first noted that an animation sequence consisting of just a few strategically positioned points of light is sufficient to create the impression in a human subject of an organism engaged in coordinated activity, such as walking. This ability to perceive biological motion has been extensively investigated, even from the perspective of development [6–11] and neurobiology [12–14].

Using conditioning procedures, several animal species have been shown to be able to discriminate between different point-light animation sequences [15–18]. Taking advantage of the learning process associated with the phenomenon of filial imprinting, Regolin et al. [19] exposed day-old domestic chicks to point-light animation sequences depicting either a walking hen or a rotating cylinder; on a subsequent free-choice test, the chicks approached the novel stimulus, irrespective of whether it was the hen or the cylinder sequence. This demonstrates that chicks, similar to other avian and mammalian species, can discriminate between point-light animation sequences. However, this tells us nothing about any possible natural predisposition of the animals to attend preferentially to biological motion stimuli.

We tested naive, newly hatched chicks, lacking any previous visual experience, to investigate whether they showed a spontaneous preference to approach stimuli depicting biological rather than non-biological motion.

**Figure 1:**
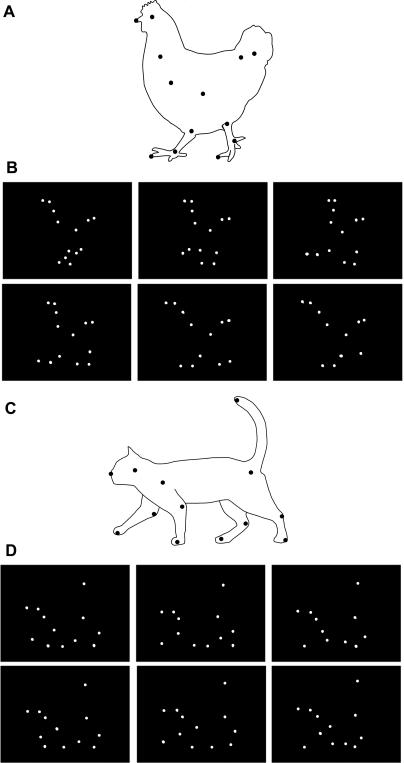
Point-Light Displays and Sample Frames from the Animation Sequences (A) The walking hen point-light display (above) The filled circles indicate the location of each point of light. (B) Six frames sampled from the walking hen animation (below) (C) The walking cat point-light display (above). (D) Six frames sampled from the walking cat animation (below).

The first point-light sequence represented a “walking hen” (13 points of light located on the digitalization of the video recording of a real animal; see Figure 1A and 1B; see also Video S1, which reproduces a version of the original walking hen stimulus). Three other sequences were used as “foil sequences.”

(1) “Rigid motion” (see Video S2 for a clip of this animation). To produce this sequence, a single frame (made of 13 points of light) from the walking hen animation sequence was randomly selected and was moved rigidly about the vertical axis so as to produce the motion of a rotating, rigid hen-like object;

(2) “Random motion” sequence (see Video S3 for a clip of this animation). In this sequence, the same set of 13 points of light described and used for each display moved now in arbitrary directions (see Materials and Methods for details about how this display was obtained).

(3) “Scrambled hen” sequence (see Video S4 for a clip of this animation). It consisted of the same set of points of light as the walking hen and the same set of frames, only now the original position of each point was spatially displaced a fixed amount throughout the animation (see Materials and Methods for more details). Every single point of light, although displaced from its original position in the walking hen animation, moved identically in this sequence to that of the walking hen. As a result, this last display no longer conveyed the perception of a hen to human observers, though it retained the appearance of biological motion of some kind of unidentified creature.

## Results/Discussion

**Figure 2:**
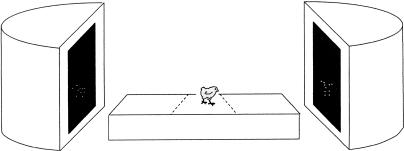
Schematic Representation of the Test Apparatus

**Figure 3:**
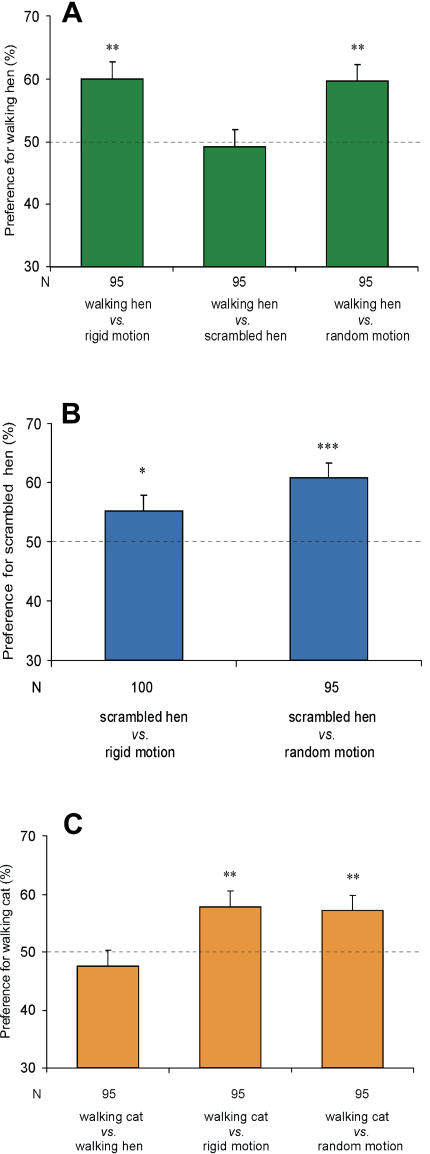
Point-Light Sequence Preferences (A) Preferences (group means and the standard error of the mean) estimated as the percentage of time spent close to the walking hen. (B) Preferences are shown as the percentage of time spent close to the scrambled hen. (C) Preferences are shown as the percentage of time spent close to the walking cat. Asterisks indicate significant departures from chance level (i.e., 50%) estimated by one-sample two-tailed *t* tests (*p < 0.05; **p < 0.01; ***p < 0.001).

Each chick underwent a 6-min free-choice test between two different displays in a standard runway apparatus (Figure 2). When presented with the walking hen and the rigid motion sequences in a free-choice test, chicks preferred to approach and stay close to the walking hen animation sequence; the same occurred when the walking hen was paired with the random motion sequence (Figure 3A). On the other hand, no preferences emerged between the walking hen and the scrambled hen sequences (Figure 3A). Analysis of variance (ANOVA) revealed a significant overall heterogeneity (F_2,279_ = 5.438, *p* < 0.005). Paired comparisons by Scheffé test revealed significant differences between the walking hen versus the rigid motion and the walking hen versus the scrambled hen conditions (*p* < 0.02), and between the walking hen versus random motion and the walking hen versus scrambled hen conditions (*p* < 0.02).

As shown in Figure 3B, the scrambled hen sequence was compared with the rigid and the random motion sequences (preferences are shown as percentage of time spent close to the scrambled hen). ANOVA did not reveal any difference between the two testing conditions (F_1,191_ = 2.239, *p* = 0.136). The scrambled hen was clearly preferred to both rigid and random motion (Figure 3B).

The results show that naive chicks exhibit clear and consistent preferences in approaching certain types of movements. Intriguingly, however, chicks' choices seemed to reflect a generic preference for the patterns of biological motion rather than a specific preference for the typical form of the hen motion. The walking hen sequence was chosen as often as the scrambled hen (Figure 3A); and both the walking hen (Figure 3A) and the scrambled hen (Figure 3B) were preferred to the rigid and random motion sequences. These findings suggest that chicks preferentially approach semi-rigid motion, the type of motion that is exhibited by vertebrate animals. In semi-rigid motion, some points maintain a fixed distance from each other (e.g., two points placed close on the same limb), but can nonetheless vary their distance with respect to other points (e.g., with respect to points located on the torso). Such a pattern of semi-rigid motion is shared by the walking and the scrambled hen sequences, even though the latter does not match any existing biological creature. As a control for this hypothesis, we used the motion of a cat (see Figure 1C and 1D; see also Video S5 for a clip of the cat animation), a species that can predate on young chicks. (This animation was obtained from the video recording of a real cat, following the procedure described for obtaining the walking hen animation). As predicted, chicks did not exhibit any preference between the walking hen and a walking cat point-light sequence, though they did prefer the walking cat to the random and the rigid motion sequences (Figure 3C). ANOVA revealed a significant overall heterogeneity (F_2,279_ =5.644, *p* = 0.004). Paired comparisons by Scheffé test revealed significant differences between the walking cat versus walking hen and the walking cat versus rigid motion conditions (*p* < 0.05), and between the walking cat versus walking hen and the walking cat versus random motion conditions (*p* < 0.05).

### Conclusion

It is known that, as a result of exposure to a particular object early in life, many species of birds and mammals will form a strong and exclusive attachment to that object, a process dubbed “filial imprinting” [2,20–23] (see also [24] for a discussion on the recent use of imprinting in order to investigate cognitive mechanisms in a comparative perspective). Motion of the object is known to facilitate the learning process [1,25,26]. However, it was not known whether any type of motion would be equally effective in eliciting approach or if specific predispositions exist for the type of motion that is most likely encountered in an animal's natural social environment. We found that visually inexperienced, newly hatched chicks, reared and hatched in darkness, at their first exposure to point-light animation sequences exhibit a spontaneous preference to approach biological motion patterns. It is likely that such a predisposition would affect the type of stimulus on which the animal is more likely to imprint on in a natural environment.

Intriguingly, the preference was not specific for the motion of a hen, but extended to the pattern of motion of other vertebrates, even to that of a potential predator, such as a cat. The predisposition found in the present research for certain kinds of movements shares characteristics in common with the predisposition for aspects of form demonstrated earlier: Visually inexperienced chicks prefer the head and neck region of a hen to artificial objects [27]. Similar to this preference for form, the preference for movement is not species specific. Evolution seems to have equipped the visually inexperienced bird with a sophisticated set of detection systems (see [28] for an extension of this argument to the human species).

The evidence of predispositions in the young chick for head and neck regions has stimulated a substantial body of work of a similar kind in our own species, concerning face recognition in the human infant (e.g., [29–31]). When considered together with our observations, these findings seem to fit a general scheme for cognitive development of recognition of the mother based on the interaction between two separate and independent systems [3,27,32–34]. The first of these systems directs the attention of the young animal toward the appropriate class of objects to learn about, in the absence of any prior specific experience (e.g., in the case of motion, toward those objects that move semi-rigidly). The second system is concerned with learning about the peculiar characteristics of the objects to which attention has been directed by the first system. Given that in a natural environment it is more likely that the newly hatched chick would encounter a mother hen rather than a cat, a developing predisposition to pay attention to objects showing the characteristic motion of vertebrates would assure highest probability to learn (by way of the imprinting mechanism) about the specific pattern of motion of the mother hen.

The perception of biological motion has been hypothesized to be an intrinsic capacity of the vertebrate visual system [5]. However, the evidence obtained so far in the human species is inconclusive: Human infants exhibit a preference for biological motion patterns starting from about 4–6 months of age [35], and this can be accounted for by both innate (maturational) and learning mechanisms. Our results with newly hatched chicks suggest that a preference for biological motion may be predisposed in the brain of vertebrates.

## Materials and Methods

### 

Eggs were incubated (using a MG 70/100 incubator) and hatched in total darkness in our laboratory. Overall, a number of 765 newly hatched chicks underwent the experiment. Each chick was tested once only for its spontaneous preference between two animation patterns: A set of 100 chicks was tested in the scrambled hen versus rigid motion comparison; we tested a set of 95 chicks for each of the other seven comparisons we investigated (i.e., walking hen versus either the random motion, solid motion, or scrambled hen sequence; scrambled hen versus the random motion sequence; and walking cat versus either the walking hen, rigid motion. or random motion sequence).

Two hours after hatching, each chick was taken from the hatchery and placed in a dark room, on a treadmill (3.7 × 10^−3^ m/s) for 30 min. Previous work [27,32] has shown that such motor activity is crucial for the development of innate predispositions in the chick. Thereafter, each chick was placed in the test apparatus, a runway measuring 80 × 20 × 20 cm (see Figure 2). At each end of the runway, a different point-light motion display was presented. The ends of the runway consisted of a transparent glass sheet (not shown in the figure,) making it visible at each end a computer screen (located 16 cm away) on which one of the two stimuli to be compared was presented.

For the purposes of this study each chick underwent the test once only (see Ethical Considerations below). The test lasted 6 min, during which time each bird could freely approach and stay by either stimulus. Using a computerized event recorder, we scored the time (in seconds) spent by each chick in either of the two 30-cm long compartments that were closer to one or the other of the two stimuli. Such raw data were thereafter computed as the overall time spent by the biological stimulus divided by the overall time spent by both the biological stimulus and the comparison stimulus combined. When the comparison was between the walking and the scrambled hen, the walking hen was arbitrarily chosen as the “biological stimulus.” Similarly, when the comparison involved the walking hen and the walking cat, the latter was arbitrarily chosen as the “biological stimulus.” Data were analyzed by ANOVA for differences between stimulus conditions; significant departures from chance level (50%) were estimated by one sample two-tailed *t* tests.

All animation sequences (see Figure 1A–D) were obtained with the use of the software program Macromedia Director (Version 6.0) and consisted of sets of 13 bright dots (95.71 candelas [cd]/m^2^) seen against a black background (0.03 cd/m^2^). Each dot was made by four pixels on a 640 × 480 pixel resolution screen; the actual visual angle measured 0° 21′ 29″ at a viewing distance of 16 cm. Animation sequences were matched for average velocity (54 pixels/s) of each of the 13 dots. Each set of points of light occupied a window of 119 × 108 pixels on the centre of the computer screen; the actual visual angle of the window measured 16° 2′ 23″ (height) and 17° 40′ 46″ (width) at a viewing distance of 16 cm.

The walking hen animation was obtained by carefully locating, frame by frame, each of the 13 points of light on the main joints of the digitalized image of the video recording of a real animal. (The same procedure was also used to produce the walking cat animation.) Twenty-three frames were required to cover an animal's entire step sequence, then the digitalized sequence was looped and projected onto a computer screen after subtraction of translation components. As a result, the display was stationary in the central window of the screen described above, but moved as if the hen was walking on a treadmill. All the other foil sequences were also produced by looping a 23-frame animation.

The scrambled hen display was obtained by consistently displacing each point of light in each frame of the walking hen sequence by 1 cm (i.e., by a visual angle of 3° 34′ 34″ at a viewing distance of 16 cm). Each point could be displaced either up, down, right, or left, at random. Although displaced compared to its position in the walking hen display, each single point of light in the scrambled hen animation retained the same motion characteristics (i.e., the same trajectory and velocity) exhibited by that point in the walking hen. As a result only the reciprocal positions of the 13 points of light differed between the walking and the scrambled hen animations. The scrambled hen display even occupied the same window on the screen as the walking hen.

The random motion display was obtained through the function “random movement and rotation” of the software program Macromedia Director MX (Version 9.0). The overall characteristics of the motion matched those portrayed in the walking hen sequence in the sense that each of the 13 points of light was associated with a different velocity, corresponding to the average velocity of each of the 13 points of light of the hen animation. Moreover the points of light in this display could move randomly within a 119 × 108 pixel window (corresponding to the area of the walking hen display); within this window, the points of light could cross each others' trajectories (which were not linear in principle of course, but being randomly determined, could assume a linear fashion for some time) and even overlap, but once they reached the edge of the defined window, they would not disappear but rather would turn around and head back. The random display, although comprising the same number of frames as the other displays, was not obtained by looping a fixed sequence of 23 frames, hence the movement in itself kept varying throughout the 6 min of presentation.

Stimuli were presented on two identical 13.8" Macintosh CRT screens with a refresh rate of 117 Hz. Apart from the light arising from the monitor screens, the room was maintained in complete darkness. (This, together with the high refresh rate of the screens, was aimed at preventing any flicker detection by the chicks).

#### Ethical considerations

All of the experiments reported comply with current Italian and European laws on the ethical treatment of animals, all experimental procedures have been licensed by the responsible office of the Italian Government (Ministero della Salute–Dipartimento Alimenti, Nutrizione e Sanità Pubblica Veterinaria), and the present project has been classified as purely behavioural testing, involving no distress or discomfort to the animals at all. Moreover, all of the chicks that entered the experiment were, after the 6-min behavioural observations, immediately caged in social groups with food and water available ad libitum and, on the second day, were donated to local farmers who provided them with free-range conditions, as approved by our Animal House licence for observational experiments on chicks.

## Supporting Information

Video S1 The Walking Hen A sample video clip of the animation employed as the walking hen stimulus. The hen is walking leftwards. This demonstration does not retain the quality of the original stimuli which were obtained in a different format.
(549 KB AVI).

Video S2 The Rotating Solid The first frame of the walking hen was treated as a solid object and rotated rigidly anticlockwise.
(13 KB AVI).

Video S3 The Random Motion A sample sequence of the stimulus employed as random motion. More details on how this stimulus was obtained can be found in the text.
(13 KB AVI).

Video S4 The Scrambled Hen The scrambled hen animation was obtained by displacing the positions of the dots of the walking hen. More information about how this was obtained can be found the text. Such manipulation results in a motion that is still perceived as biological, although it does not belong to any particular known animal.
(15 KB AVI).

Video S5 The Walking Cat A sample video clip of the animation employed as walking cat stimulus. The cat is heading to the left.
(14 KB AVI).

## Abbreviations

ANOVA: analysis of variance
cd: candela

## References

[pbio-0030208-b01] Bateson PPG, Heyes C, Huber L (2000). What must be known in order to understand imprinting?. The evolution of cognition.

[pbio-0030208-b02] Horn G (2004). Pathways of the past: The imprint of memory. Nat Neurosci.

[pbio-0030208-b03] Morton J, Johnson MH (1991). CONSPEC and CONLERN: A two process theory of infant face recognition. Psychol Rev.

[pbio-0030208-b04] Bolhuis JJ (1991). Mechanisms of avian imprinting: A review. Biol Rev.

[pbio-0030208-b05] Johansson G (1973). Visual perception of biological motion and a model for its analysis. Percept Psychophys.

[pbio-0030208-b06] Arterberry ME, Bornstein MH (2002). Infant perceptual and conceptual categorization: The role of static and dynamic stimulus attributes. Cognition.

[pbio-0030208-b07] Bertenthal BI, Proffitt D, Kramer S (1987). Perception of biomechanical motion by infants: Implementation of various processing constraints. J Exp Psychol Hum Percept Perform.

[pbio-0030208-b08] Bertenthal BI, Proffitt DR, Spetner NB, Thomas MA (1985). The development of infant sensitivity to biomechanical motions. Child Dev.

[pbio-0030208-b09] Frith U, Frith CD (2003). Development and neurophysiology of mentalizing. Phil Trans R Soc Lond B Biol Sci.

[pbio-0030208-b10] Schmuckler MA, Fairhall JL (2001). Visual proprioceptive intermodal perception using point light displays. Child Dev.

[pbio-0030208-b11] Pavlova M, Krageloh-Mann I, Sokolov A, Birbaumer M (2001). Recognition of point-light biological motion displays by young children. Perception.

[pbio-0030208-b12] Grossman E, Donnelly M, Price R, Pickens V, Morgan V (2000). Brain areas involved in perception of biological motion. J Cogn Neurosci.

[pbio-0030208-b13] Oram MW, Perrett DI (1994). Responses of anterior superior temporal polysensory (STPa) neurons to “biological motion” stimuli. J Cogn Neurosci.

[pbio-0030208-b14] Vaina L, Solomon J, Choudhury S, Sinha P, Belliveau JW (2001). Functional neuroanatomy of biological motion perception in humans. Proc Nat Acad Sci U S A.

[pbio-0030208-b15] Blake R (1993). Cats perceive biological motion. Psychol Sci.

[pbio-0030208-b16] Dittrich WH, Lea SEG, Barrett J, Gurr PR (1998). Categorization of natural movements by pigeons: Visual concept discrimination and biological motion. J Exp Anal Behav.

[pbio-0030208-b17] Omori E, Watanabe S (1996). Discrimination of Johansson's stimuli in pigeons. Int J Comp Psychol.

[pbio-0030208-b18] Perrett DI, Harries MH, Benson PJ, Chitty AJ, Mistlin AJ, Blake A, Troscianko T (1990). Retrieval of structure from rigid and biological motion: An analysis of the visual responses of neurones in the macaque temporal cortex. AI and the eye.

[pbio-0030208-b19] Regolin L, Tommasi L, Vallortigara G (2000). Visual perception of biological motion in newly hatched chicks as revealed by an imprinting procedure. Anim Cogn.

[pbio-0030208-b20] Lorenz K (1937). The companion in the bird's world. Auk.

[pbio-0030208-b21] Bateson PPG (1966). The characteristics and context of imprinting. Biol Rev Camb Philos Soc.

[pbio-0030208-b22] Sluckin W (1972). Imprinting and early learning.

[pbio-0030208-b23] Horn G (1998). Visual imprinting and the neural mechanism of recognition memory. Trends Neurosci.

[pbio-0030208-b24] Wasserman EA, Zentall TR, Vallortigara G (2005). The cognitive chicken: Visual and spatial cognition in a non-mammalian brain. Comparative cognition: Experimental explorations of animal intelligence.

[pbio-0030208-b25] Horn G (1985). Memory, imprinting and the brain.

[pbio-0030208-b26] Rogers LJ (1995). The development of brain and behaviour in the chicken.

[pbio-0030208-b27] Johnson MH, Horn G (1988). Development of filial preferences in dark-reared chicks. Anim Behav.

[pbio-0030208-b28] Spelke ES, Gentner D, Goldin-Meadow S (2003). What makes us smart. Core knowledge and natural language. Language in mind. Advances in the study of language and thought.

[pbio-0030208-b29] Johnson M (1992). Imprinting and the development of face recognition: From chick to man. Curr Dir Psych Sci.

[pbio-0030208-b30] Johnson MH, Morton J (1991). Biology and cognitive development. The case of face recognition.

[pbio-0030208-b31] Turati C, Simion F, Milani I, Umiltà C (2002). Newborns' preference for faces: What is crucial?. Dev Psychol.

[pbio-0030208-b32] Johnson MH, Bolhuis JJ, Horn G (1985). Interaction between acquired preferences and developing predispositions during imprinting. Anim Behav.

[pbio-0030208-b33] Horn G, McCabe B (1984). Predispositions and preferences. Effects on imprinting of lesions to the chick brain. Anim Behav.

[pbio-0030208-b34] Johnson MH, Horn G (1986). Dissociation of recognition memory and associative learning by a restricted lesion of the chick forebrain. Neuropsychologia.

[pbio-0030208-b35] Fox R, McDaniel C (1982). The perception of biological motion by human infants. Science.

